# Rapamycin Augmentation of Chronic Ketamine as a Novel Treatment for Complex Regional Pain Syndrome

**DOI:** 10.7759/cureus.43715

**Published:** 2023-08-18

**Authors:** Ayush Sharma, Maral Tajerian, Jon Berner

**Affiliations:** 1 Pain Management, Woodinville Psychiatric Associates, Woodinville, USA; 2 Department of Biology, Queens College, City University of New York, Flushing, USA; 3 The Graduate Center, City University of New York, New York, USA; 4 Psychiatry, Woodinville Psychiatric Associates, Woodinville, USA

**Keywords:** autoantigen, nfatc4, microglia, treg, crps, rapamycin, ketamine

## Abstract

This case report describes the dramatic clinical response of refractory chronic complex regional pain syndrome to combined immunomodulatory treatment. Ketamine and rapamycin markedly minimized pain historically associated with suicidal behavior, increased baseline activity, and allowed for a reduction in palliative polypharmacy. The piecewise mechanism of action is unclear given multiple postulated targets, such as microglia, astroglia, T-regulatory cells, B-regulatory cells, or neurons. Relevant laboratory and genetic information may allow the application of this treatment to other affected individuals.

## Introduction

Complex regional pain syndrome (CRPS) is a chronic pain condition that unpredictably develops following injury [1]. The pain is disproportionate to injury in magnitude, often leading to disability and, in severe cases, to consideration of and/or completed suicide. CRPS pain, most frequently at the site of injury, or less frequently systemically, is associated with hyperalgesia, temperature and color change in the skin, sweating or swelling, and non-specific movement abnormalities. Although the Budapest criteria for CRPS has served a useful purpose in directing basic and translational research toward a homogeneous patient population, it is likely that this syndrome is embedded in a larger complicated clinical and pathophysiological landscape of related neurogenic pain syndromes. Recent genome-wide association studies of 380,000 patients with chronic pain revealed substantial polygenetic heritability and comorbidity with psychiatric, autoimmune, and anthropometric traits [2].

Causal pathophysiological mechanisms continue to be poorly understood. Current research is slowly attempting to apply lessons learned from the biphasic acute injury and subsequent repair response observed in peripheral tissues over time to analogous stromal (microglia and astroglia) and parenchymal (neurons) tissue in the nervous system [3].

Current palliative treatments for CRPS are very diverse but too often only partially effective, likely consistent with the phenotypic diversity observed within a boolean diagnostic category. These range from conservative psychological and physical therapies, medications (steroids, cannabinoids, norepinephrine reuptake inhibitors, opioids, bisphosphonates, hydroxychloroquine), or minimally invasive treatments such as sympathetic ganglion blocks or spinal cord stimulators [4].

Although the commonly used anesthetic, ketamine, is increasingly recommended as the treatment of choice for refractory CRPS [5], many patients have limited access to tertiary centers with expensive and inconvenient periodic intravenous (IV) infusions. Although some have recommended that self-administered oral or intranasal ketamine is a practical alternative to IV ketamine, in our experience, most providers are uncomfortable managing addiction and delirium side effects associated with these routes of administration [6].

Recent research in a common syndromic disorder, major depression, sharing close genetic overlaps with neurogenic pain [2], suggests profound synergistic effects when ketamine is co-administered with rapamycin [7]. Although at the time, the authors struggled with a mechanistic model for this finding, with previous in vitro work describing additive effects on suppression of cytokine release from macrophages stimulated with lipopolysaccharide (LPS) [8]. These two research findings suggest additional clinical attention should be directed toward downregulating pathological immunological signals between neurons and supporting stromal tissue.

We applied these recent research findings to a patient with partial control of CRPS symptoms maintained on 150 mg intranasal ketamine daily. Although she was not imminently suicidal, she was profoundly disabled and was passively waiting for death despite aggressive psychiatric treatment with psychotherapy, antidepressants, and electroconvulsive therapy (ECT). After a review of these recent research findings, this patient consented to a trial of this combination treatment under the Washington State Right to Try Act (Chapter 69.77 RCW). Subsequent to this trial, the patient provided written consent for the release of her case history (Appendices).

## Case presentation

Disease progression and past interventions

The patient is a 45-year-old Caucasian female. At age 29, a car accident resulted in the onset of CRPS, characterized by a two-month acute phase of whiplash, followed by a chronic phase including occipital neuralgia and bilateral atypical trigeminal neuralgia consistent with temporomandibular joint (TMJ) pain. At age 31, the patient was clinically managed as having a CRPS variant although vasomotor and sudomotor symptoms were not noted in the historical record. Dystonia and burning pain disproportionate to normal imaging partially responded to bilateral stellate and lumbar sympathetic blocks. Two more car accidents at age 33 and 35 caused a flare of acute symptoms, as well as a broken right ankle and a thoracic spine injury resulting in radiating pain to the ribcage, sternum, diaphragm, and arms. Trigger point injections were administered to the spinous process of the affected vertebrae, and additional overnight intravenous injections of ketamine (50-200 mg/hour) provided only some relief for the thoracic pain. Full CRPS developed in the right ankle/foot area with swelling, red and blue color change, cold sensations, and dystonia; slow *mirror* manifestations occurred in the left foot over time. Spinal cord stimulators were inserted at ages 36 and 41, providing major pain relief to the foot; however, the patient still could only stand for two hours a day. Palliative treatment with botulinum toxin, trigger point injections, physical therapy, chiropractic manipulation, hyperbaric oxygen, and paraspinal and occipital nerve injections had no sustained effect on long-term disability. Medications including opioids, benzodiazepines, gabapentinoids, norepinephrine reuptake inhibitors, tizanidine, baclofen, and sodium oxybate had no sustained effect on long-term disability. Intranasal ketamine after 2018 provided some improved pain control when she was not in a full flare but was unable to affect long-term disability.

The duration and intensity of the pain from the thoracic flares progressed with age. At age 36, she had a prolonged flare with subsequent three suicide attempts over the span of three months. Multiple inpatient admissions for prolonged ketamine infusions were ineffective. Given prolonged neurological sequelae from her third attempt (diplopia), the patient pursued and obtained authorization for assisted suicide under the Washington State Death with Dignity Act. Two separate physicians, distinct from the treatment team, provided this authorization. Although suicidal behavior is a defining criterion for the syndrome of depression, the patient historically poorly met the criteria for the full syndrome. Her affect with her providers was full and largely congruent with her medical circumstances. Her cognitive style was pragmatic, completely without catastrophic reactions and/or overvalued ideas frequently seen with autism traits. No one in the historical medical record had substantial concerns regarding impaired competence to engage in aggressive medical interventions.

Table 1 presents the Budapest criteria for CRPS. Table 2 summarizes the time course of the patient’s experience with CRPS including significant events, treatments, and outcomes.

**Table 1:** Budapest criteria for CRPS. The current protocol for clinical CRPS diagnosis. Patients must report at least one symptom in each category and must display signs of at least one symptom in two or more categories at the time of evaluation. Adapted from Goebel et al. [9]. CRPS: complex regional pain syndrome

Category	Symptoms
Sensory	Hyperalgesia, allodynia
Vasomotor	Temperature asymmetry, skin color change/asymmetry
Sudomotor	Edema, sweating changes, sweating asymmetry
Motor/trophic	Decreased range of motion, motor dysfunction (weakness, tremor, dystonia), trophic changes (hair, nail, skin)

**Table 2:** Timeline of CRPS progression and treatment methods. Timeline of patient care including relevant disease progression and treatments with outcomes. CRPS: complex regional pain syndrome; TMJ: temporomandibular joint

Date	Associated event
2006	The first car accident caused the onset of CRPS symptoms (acute whiplash, chronic TMJ pain)
2010	The second car accident caused a flare-up of previous CRPS and a broken right ankle
2012	The third car accident caused a flare-up of previous CRPS symptoms and thoracic spine injury, resulting in radiating pain to the ribcage, sternum, diaphragm, and arms.
2012	Trigger point injections were administered to the spinous process of affected vertebrae, providing some alleviation of thoracic pain
2012	Overnight intravenous injections of ketamine provided some relief for the thoracic pain but not ankle/foot pain
2013	The spinal cord stimulator provided major pain relief to the foot; however, major deficits in locomotive ability and hypersomnia persisted
2013	Severe CRPS flare led to three suicide attempts within three months. The patient obtained authorization for assisted suicide but did not follow through, citing improvement in pain severity
2018	The spinal cord stimulator was replaced without major changes in the disease state. Ketamine was titrated to 150 mg daily by the intranasal route
May 2022	Daily orally administered rapamycin (1 mg) and intranasal ketamine (150 mg) began. The patient’s energy levels radically increased, but the effects subsided within three weeks
June 2022	The rapamycin dose was increased to 2 mg. Prior energy level improvements restored
August 2022	The rapamycin dose was increased to 3 mg. The patient reported a significant decrease in both the length of CRPS flares and the degree of pain

Relevant laboratory and genetic information

During her relentless downward course, the patient provided multiple relevant laboratory findings, including absent slow-wave sleep on polysomnography in 2008 and 2010, antidiabetic serum metabolomics (HgA1c: 4.4 and valine/glycine ratio: 184/443), T-lymphocyte insensitivity to growth arrest induced by glutamine restriction (61% of control when glutamine is present, >5% of upper limit of normal), serum mitochondrial abnormalities (low Q10 and carnitine), *ryanodine receptor 2* (*RYR2*) mutation with whole exome sequencing, cerebrospinal fluid metabolome purine abnormalities (guanosine z-score: 2.2, adenosine z-score: 2.1, adenosine monophosphate was uniquely present), and buccal epithelium respirometry with absent complex 1 activity.

Introduction of rapamycin and ketamine

At age 44, the patient began a daily 1 mg orally administered rapamycin treatment along with 150 mg of intranasal ketamine. After four days, the patient’s energy levels and pain relief dramatically improved. Her ability for moderate physical activity increased to over 10 hours per day (14K steps) from a baseline of 15 minutes (400 steps), and sleep was adequate at nine to ten hours per night. Three weeks after the first administration, pain relief and energy decreased to a new baseline, still slightly improved from the pre-rapamycin state. One month later, the rapamycin dose was increased to 2 mg per day, and after one week, all prior improvements returned, with an even higher energy level reported than before. One month later, the dose was again increased to 3 mg similar to mean dosing in systemic lupus erythematosus (SLE). A major improvement was seen in CRPS flare-ups from accidental bumps and falls: the time course of each flare fell from three to six months to just one week on average, and the pain level was self-reported as decreased by 50%. Adherence and tolerability of the treatment plan were self-assessed, while tangible increases in energy levels were assessed using smart watch step counting.

Patient perspective

The following statement was provided by the patient regarding her experience with CRPS and associated treatments:

What was initially thought to be a mild case of whiplash after being rear ended, turned into a 16-year and counting chronic pain nightmare. I’ve tried almost everything medically possible to get any amount of relief, while still always hoping for a cure.

*The first two years weren’t very bad. The pain was confined to my upper body. A TMJ specialist*’*s treatment, with upper and lower orthotics, helped ease my head pain by approximately 50%, and seeing a chiropractor, massage therapist, and physical therapist, each one to three times a week, kept my pain below a 5/10, otherwise being up to an 8/10. That was until an old low back injury flared up two years after the car accident, causing the next two years to be a nightmare. Oral ketamine helped calm my pain down to around a 6/10. I eventually turned to an anesthesiologist, who did cervical stellate ganglion blocks, once per week for three weeks, each helping 20-30% initially, reverting to 5% after a few weeks. Over the course of a year, I had approximately 10 rounds of those (30 total), by gaining 5% of pain relief each round, those had helped relieve 80% of my upper body chronic pain, combined with the relief the oral ketamine gave me. I was close to being pain-free, then was rear-ended again.*

This accident broke my right ankle, which took one and a half years to diagnose and a surgery to repair, caused severe CPRS in both ankles and feet. Eventually, a spinal cord stimulator implant was 80-90% successful in treating my ankle and foot pain, though didn’t help the now-flared upper body pain and caused the severe thoracic spine pain that a third car accident had caused to become much worse. Over 22 months, I had seven 10/10 flare ups. Intravenous ketamine drips at this time provided slight relief, but seeing no end in sight, I tried three times to take my life by medication overdoses that would have been successful had my husband not found me, and almost followed through with physician-assisted suicide. I continued with life and my previous therapies, however, I had to use a motorized scooter just to get around the house for two years and couldn’t walk over 200 feet by myself.

Eventually, I was introduced to rapamycin. It has been a game changer. By far the most effective treatment I’ve had. It’s the only thing that’s helped the low back/left leg chronic pain which is now around a 2/10 pain level. In all, I’ve had over 2,000 medical appointments. I have tried over 25 medications that had no effect. I had and continue to find some relief from certain medications like muscle relaxants, synthetic opioids, anxiety medications, and antidepressants. Having been on rapamycin for about 10 months, I’ve been able to discontinue an antidepressant, almost discontinue another, cut my muscle relaxant dose in half, and am hoping to discontinue more as my progress continues. Rapamycin truly turned my life around and I can’t imagine living my life again as I did prior to starting it.

## Discussion

This history suggests that rapamycin, either singly or in conjunction with ketamine, provided dramatic sustained improvement in a single case of chronic severe refractory CRPS.

The precise targets of these agents remain unclear. The mechanistic target of rapamycin, mTORC1, is present in every cell in the nervous system. mTORC1 is a master regulator of protein transcription rates, via S6K and 4EPB1, typically inactive during starvation/intracellular nutrient or ATP depletion and active during glycolytic cell growth and division. The canonical target of ketamine, the NMDA receptor, is present in both neurons and resident microglia, but noncanonical targets on other cells are also likely.

Tissue-resident glia

Tissue-resident microglia, uniquely derived from the embryonic yolk sac as distinct from peripheral macrophages, is one possible target of this combination, although direct evidence from preclinical models of CRPS is scarce. In vitro, LPS-induced inflammatory microglia treated with ketamine showed a decreased proinflammatory cytokine flux (tumor necrosis factor-alpha (TNF-α), interleukin-1β (IL-1β)) and nitrous oxide production [10], suggesting that ketamine can downregulate the microglial inflammatory response [3,11]. In mice, constitutively active TOR1 activity has been shown to result in broad defects in the availability of anti-inflammatory microglia [12]. Consistent with the above, rat models have shown that rapamycin downregulates microglial expression of cytokines TNF-α and IL-1β [13].

Astrocyte function is modulated by microglia activity. Proinflammatory microglia can also induce proinflammatory astrocyte activation mediated by IL-1α, TNF, and C1q [14], which may suggest that activated astroglia resulting from active microglia may play a role in the CRPS disease phenotype. However, this cytokine-facilitated astrocytic activation can be reversed by increases in the anti-inflammatory cytokine transforming growth factor-beta, whose downregulation in the prefrontal cortex was restored by (R)-ketamine in a mouse model of depression [15]. In a preclinical model of CRPS in mice, ultra-low-dose systemic ketamine was linked to pain relief and the normalization of increased astrocytic numbers in the dorsal horn of the spinal cord [3].

Lymphocyte contributions

Tissue-resident T-regulatory lymphocytes (Tregs) are also a potential target of rapamycin. Although the brain and spinal cord are generally immunologically privileged with very low concentrations of resident Tregs, it appears that even marginal increases in brain-resident or circulatory Treg activity can have profound effects on baseline microglial baseline activity and astrogliosis [16]. It can also modulate the autoimmune response [17]. The clinical literature, in turn, suggests excellent efficacy for rapamycin in SLE [18] and multiple sclerosis [19] with documented induction of increased serum Treg proportions.

B-lymphocytes are also a target of ketamine and rapamycin. The antagonism of NMDA receptors on these cells inhibits antibody effector functions and induces IL-10 production downregulating local antigen-presenting cells and inducing antigen-specific Treg activation [20]. Isolation of pro-nociceptive autoantigens from the sera of both humans and animals suggests some degree of causal innate immunity from activated B-cells [21-23]. However, precise mechanisms need further study. IgM species appear to dominate relative to IgG in animal models and certain translational studies [24], suggesting a relatively smaller contribution from the T-follicular helper subtype despite evidence for localized lymphadenopathy after fractures associated with CRPS. The localized nature of pain observed in many patients despite generalized serum autoantibodies potentially directs attention to local tissue-resident Tregs as mechanisms for limiting the appearance of chronic widespread pain seen in fibromyalgia-like neurogenic pain syndromes. The literature on SLE provides some insights into the potential to regulate autoantibodies, where clinical improvement associated with rapamycin use includes marked reductions in IgA and IgG anti-beta-2-glycoprotein at one month but no effect on IgG levels over 12 months. Rapamycin insensitivity of memory B-cells producing IgG in SLE stands in stark contrast to the extreme sensitivity of memory CD8 cells to rapamycin. Relative depletion of these cells defines rapamycin response (area under the curve of 0.967) [25]. This disjunction of the rapamycin effect is clearly seen in cell culture in terms of mTORC1-independent protein kinase B (AKT) and mTORC2 activity observed in B-lymphocytes related to T-lymphocytes [26,27].

Parenchymal neurons

Neuronal tissue is also a potential anti-inflammatory target of ketamine. In a mouse model of CRPS, the chronic stage of pain was associated with decreased levels of glutamate receptor subunit NMDAR2b in the spinal cord, with ketamine exposure being linked to increased receptor expression in both the CRPS and control groups [3]. This seemingly paradoxical result may indicate that the efficacy of ketamine may not be directly due to NMDA receptor blockade on neurons. It is noteworthy that NMDAR2b levels appear to be unchanged in female mice with CRPS, thereby signaling sex differences in disease progression [28].

In a different context, ketamine blocks depression associated with systemic LPS exposure by downregulating the nuclear factor of activated T-cells 4 (Nfatc4) in the prefrontal cortex [29]. Further experimental work suggests a mechanistic explanation related to the induction of the M2 microglial phenotype by the secretion of Nfatc4-mediated IL-4 associated with low-dose but not high-dose NMDA activation [30].

Consideration of patient phenotype

The correspondence of these postulated mechanisms to the complex tissue phenotypes observed in this patient is beyond the scope of this brief clinical paper. Nevertheless, this history underscores the near certainty that the full CRPS requires multiple hits in distinct pathways, perhaps consistent with clinical experience with incomplete responses to diverse palliative interventions. We note the diversity and richness of the literature related to the phenotypic presentations: increased serum butyrate availability from the microbiome has direct effects on downregulating calcium flux via RYR2 and upregulating anti-inflammatory macrophage populations in the dorsal root ganglion [31], PI3K gain of function is associated with immune hyperactivity [32] and insulin sensitivity [33] (valine to glycine ratio), purine microgradients are created in the central nervous system (CNS) from tissue injury and induce microglial activation, acting as a *find me signal* [34], and impaired mitochondrial respiration (high glycolytic flux) biases T-cell responses to effector subtypes and macrophage proinflammatory secretory activation [35].

Recent work examining glutamine synthetase gain of function as a determinant of ovarian cancer aggressiveness potentially very closely corresponds to the patient’s observed insensitivity to glutamine depletion-restriction of T-cell proliferation [36]. In the cancer model, glutamine availability in the stromal ovarian cell determines the equilibrium ratio of tissue *M1-like*/*M2-like* tissue-resident macrophage via tonic levels of N-acetylaspartate (NAA). In this model, NAA rather than extracellular glutamate controls basal NMDA receptor activity in *M1-like* macrophages. We have observed consistent with the above in our clinical population that a glutamine sensitivity in human T-cells correlates with ketamine response.

Dose finding in a two-dimensional landscape with axes by defined ketamine and rapamycin dose

Although it may superficially appear that the bulk of this patient’s improvement was attributed to rapamycin, we hesitate to suggest that future studies of CRPS and neurogenic pain should solely investigate rapamycin alone in monotherapy. The tissue response to rapamycin alone is highly nonlinear in a single in-vitro model defined by cytokine release after LPS challenge [8]: low-dose rapamycin increases inflammatory cytokine production presumably via increased 4EBP1-dependent translation in contrast to marked decrements at higher dosages. Concurrent ketamine in this model blocks this excessive cytokine release. We have seen in our clinical population uncommon, but not rare, inflammatory symptoms induced by starting rapamycin in monotherapy at 1 mg daily. Conversely, pathological macrophage *M2-like* MKi-67 proliferation induced by NMDA blockage on *M1-like* macrophages by the endogenous ligand NAA may be modulated by the concurrent use of rapamycin [36].

The previous data complicates the design of necessary future randomized placebo-controlled trials. Starting at the origin of this landscape (ketamine dose 0, rapamycin dose 0) may represent an unfavorable local minimum. Temporal path dependence is likely present in some cases, suggesting the initial use of ketamine titrated to the maximally tolerated dose before the initiation of rapamycin. Alternative gradient descent models for dose finding can also be considered, potentially defining the origin as the mean population dosing for ketamine (4.2 mg/kg PO in our clinic) and for rapamycin, e.g., 3 mg in active SLE [25].

Generalizing from a single case study to a larger population with substantial phenotypic diversity despite a superficially unitary research diagnosis, i.e., CRPS, will require slow and expensive recruitment and monitoring of large clinical populations. This case study suggests the presence of numerous dimensionally distributed biomarkers which might allow expedited population recruitment, smaller sample sizes, and more objective markers guiding gradient descent during dose finding with ketamine alone, rapamycin alone, or in combination.

Limitations of rapamycin and ketamine

Patient selection, therapeutic dose titration, and long-term risks associated with this combination are poorly understood. Grounds for optimism can be gathered from the animal longevity literature where rapamycin may have the most robust and largest effect size of all agents currently under consideration. On the other hand, clinical literature and our experience suggest certain individuals on rapamycin will be vulnerable to opportunistic infections and sepsis [37], insulin resistance [38], poor acute wound healing [39], and capillary leak syndromes [40]. Chronic self-administered ketamine use also poses a risk of cystitis [41] and addiction, although incidence rates have been low in our experience and possibly mitigated by concurrent rapamycin. In vitro data additionally suggests that combination treatment with ketamine and rapamycin may have augmented immunosuppressive effects on *M1-like* macrophage activation with implications for viral, cancer, and intracellular bacteria defense mediated by the TH1 CD4+ lymphocyte population [8,36].

CRPS has profound effects on CNS function, potentially persistent similar to memory B-cells and T-cells [42]. Although increased efficiency of synaptic pruning and synaptic trophic support associated with optimal *M2-like* microglia activity is theoretically curative of the cognitive defects observed in CRPS and perhaps its genetically related cousin post-traumatic stress disorder [2], optimal activity is poorly defined in the individual patient at an individual point in time. Caution is warranted by the observed and often clinically limiting neurotoxicity of cyclosporine. Doses high enough to suppress acquired immunity in transplant rejection may impair physiological production on IL-4 modulation of M2 microglia [30]. In our experience, optimal dosing of chronic ketamine is highly variable across individuals.

## Conclusions

This case report provides a conceptual framework for the application of rational polypharmacy to central pain syndromes. In the absence of technical abilities to obtain definitive tissue biopsies, until recently, chronic pain was often characterized in the *biopsychosocial model* as largely driven by poorly defined *stress* rather than reductionist medical language. New technical advances and estimation of the patient’s individual gain of function variation in metabolism and intracellular calcium dynamics may allow for the precise downregulation of an immune system overly reactive to neoantigens generated during sterile tissue injury.

## Figures and Tables

**Figure 1:**
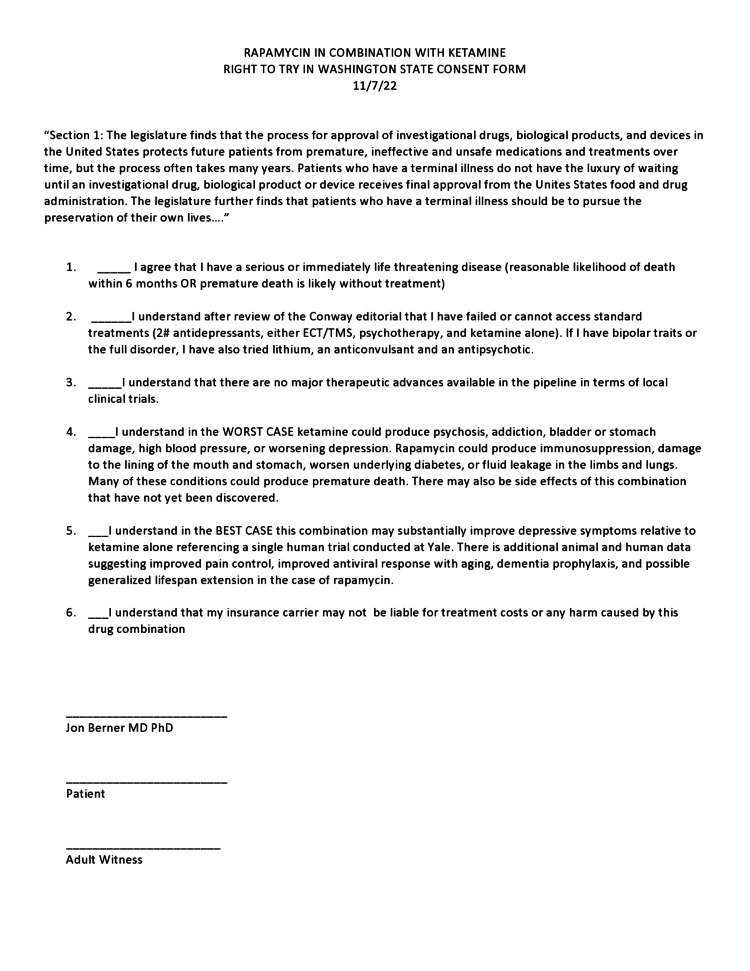
Rapamycin and ketamine right to try consent form.
